# Epigenome‐wide DNA methylation analysis of myasthenia gravis

**DOI:** 10.1002/2211-5463.13656

**Published:** 2023-06-10

**Authors:** Jingjing Lin, Linshuang Tao, Lu Deng, Ruyi Zhou, Shuyue Lou, Songfang Chen, Xuanyu Chen, Chunxing Lu, Peijun Li, Beilei Hu

**Affiliations:** ^1^ Department of Neurology The Second Affiliated Hospital and Yuying Children's Hospital of Wenzhou Medical University China; ^2^ Department of Nephrology Taizhou First People's Hospital, Affiliated Huangyan Hospital of Wenzhou Medical University China

**Keywords:** DNA methylation, epigenetics, high throughput, myasthenia gravis, neuromuscular junction

## Abstract

Myasthenia gravis (MG) is a common neuromuscular junction disorder and autoimmune disease mediated by several antibodies. Several studies have shown that genetic factors play an important role in MG pathogenesis. To gain insight into the epigenetic factors affecting MG, we report here genome‐scale DNA methylation profiles of MG. DNA was extracted from eight MG patients and four healthy controls for genome‐wide DNA methylation analysis using the Illumina HumanMethylation 850K BeadChip. Verification of pyrosequencing was conducted based on differential methylation positions. Subsequently, C2C12 and HT22 cell lines (derived from mouse) were treated with demethylation drugs. Transcribed mRNA of the screened differential genes was detected using quantitative real‐time PCR. The control and MG group were compared, and two key probe positions were selected. The corresponding genes were *CAMK1D* and *CREB5* (*P* < 0.05). Similarly, the myasthenic crisis (MC) and non‐MC group were compared and four key probe positions were selected. The corresponding genes were *SAV1, STK3, YAP1*, and *WWTR1* (*P* < 0.05). Subsequently, pyrosequencing was performed for verification, revealing that hypomethylation of CAMK1D was significantly different between the MG and control group (*P* < 0.001). Moreover, transcription of *CREB5, PKD, YAP1*, and *STK3* genes in the C2C12 cells was downregulated (*P* < 0.05) after drug treatment, but only *YAP1* mRNA was downregulated in HT22 cells (*P* < 0.05). This is the first study to investigate genome‐scale DNA methylation profiles of MG using 850 K BeadChip. The identified molecular markers of methylation may aid in the prevention, diagnosis, treatment, and prognosis of MG.

## Graphical abstract

Myasthenia gravis (MG) is a neuromuscular junction disorder. Here, genome‐wide methylation analysis was performed using the Illumina HumanMethylation 850K BeadChip. The verification of pyrosequencing and cell culture *in vitro* was conducted based on differential methylation positions. This study may provide useful information for the study of pathogenesis and pathological mechanism of MG.

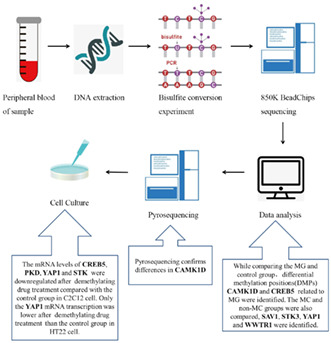

**Abbreviations**
AChRs: acetylcholine receptors
DAC: 5‐aza‐2′‐deoxycytidine
DMP: differential methylation position
DMR: differential methylation region
FEM: functional epigenetic modules
GO: Gene Ontology
HLA: human leukocyte antigen
KEGG: Kyoto Encyclopedia of Genes and Genomes
MC: myasthenic crisis
MG: myasthenia gravis
NMJ: neuromuscular junction
PCA: principal component analysis
PPI: protein interaction network
SVD: singular value decomposition analysis

Myasthenia gravis (MG), a common neuromuscular junction (NMJ) disorder, is an autoimmune disease mediated by several antibodies. Clinical features of MG mainly include fluctuating muscle weakness and fatigability after an activity. MG often affects the extraocular muscles, bulbar muscles, and the proximal limb muscles. Around 15–25% of MG patients will have myasthenic crisis (MC), which causes respiratory muscle weakness and respiratory failure, increasing the risk of death. According to reports, the annual incidence of MG in southern China is estimated to be 1.55–3.66 per 100 000, and the prevalence is estimated to be 2.19–11.07 per 100 000 [1]. Current studies have shown that genetic susceptibility, autoimmune environment, and autoantibodies participate in the pathogenesis of NMJ disorder. Moreover, the above factors affect the production of endplate potential and muscle contraction causing MG [2]. Many studies have shown that autoantibodies against normal body antigens, including acetylcholine receptors (AChRs), muscle‐specific kinase, and lipoprotein‐related protein 4, are related to the development of MG [3]. Genetic factors are also one of the important factors for MG development. Moreover, cytotoxic T lymphocyte correlated antigen‐4 (CTLA4) gene, the human leukocyte antigen (HLA) gene, the cholinergic receptor nicotinic alpha 1 (CHRNA1) gene, and the protein tyrosine phosphatase nonreceptor type 22 (PTPN22) gene participate in MG development [4, 5, 6]. With the development of detection methods, the role of epigenetics in diseases has gradually been valued.

DNA methylation is one of the most common phenomena of epigenetic alterations. DNA methylation is a form of chemical modification of DNA, which alters RNA transcription without changing the DNA sequence. Abnormal DNA methylation is one of the most extensive epigenetic changes in cancer development and can alter gene expression [7, 8]. Changes in DNA methylation are heritable and affect the active state of the X chromosome, and gene expression. Mamrut *et al*. compared the transcriptome and methylome of peripheral monocytes in female twins with MG. The results showed that alteration of gene expression or methylation level may jointly cause MG [9], suggesting that genetic susceptibility may contribute more to the pathogenesis of MG than previously thought.

In this research, the epigenetic DNA methylation pattern in MG patients and healthy controls, and the differentially methylated positions (DMPs) were identified. The new potential biological functions of genes are proposed through bioinformatics tools analysis.

## Methods

### Ethical considerations

This study was conducted strictly in accordance with the Helsinki Declaration and was approved by the permission Ethics Committee of the Second Affiliated Hospital of Wenzhou Medical University and Yuying Children's Hospital (LCKY2018‐12). In addition, patients provided informed consent to participate in this study in writing.

### Participants and design

The study participants received treatment at the Department of Neurology, the Second Affiliated Hospital of Wenzhou Medical University and Yuying Children's Hospital. The patients were diagnosed by two neurologists at least at the deputy director level in accordance with the 2015 China Myasthenia Gravis Diagnosis and Treatment Guidelines. The diagnostic criteria were as follows: (a) with muscle weakness of some specific striated muscles, with mild symptoms in the morning but severe in the afternoon; (b) positive for the neostigmine test; (c) positive for the repetitive nerve stimulation; and (d) with AChR antibodies in the peripheral blood of most patients with generalized MG. Basing on the typical clinical characteristics MG, has pharmacological and/or neurophysiological characteristics, and can be diagnosed in the clinic. Qualified units can detect anti‐AChR antibodies in the blood of patients for further verification of the diagnosis. In summary, the diagnosis was done if the above first point and one of 2, 3, 4 points on selection criteria were met. Patients with other diseases, such as Lambert–Eaton myasthenic syndrome, progressive bulbar palsy, botulism, and metabolic myopathy, were excluded. The MC diagnostic criteria were as follows: sudden increase in muscle weakness, progressive weakness, or paralysis of the respiratory and swallowing muscles, all of which are life‐threatening.

### DNA methylation experiment

DNA was extracted from the peripheral blood of the patients using the DNeasy Blood and Tissue Kit (Qiagen, Beijing, China). The purity and concentration of DNA were measured using Nanodrop 2000 (Thermo, Beijing, China). A concentration of 500 ng DNA of each sample was converted by bisulfite using EZ DNA Methylation Kits (Zymo Research, Tustin, CA, USA). The converted products were loaded into 850K BeadChips in accordance with the manufacturer's guide and protocol (Illumina, Hayward, CA, USA). The chip detects more than 850 000 sites in each sample, comprehensively covering CpG islands, promoters, coding regions, etc. The whole processing steps related to chips, such as amplification, labeling, cleaning, hybridization, and scanning, were performed by Shanghai Pudong Decode Life Science Research Institute.

### Data analysis

The data were analyzed using champ [10] package in r. The β value was used to represent the proportion of each methylated CpG position. Firstly, probes with detection *P*‐value < 0.01, probes with < 3 beads in at least 10% of samples, non‐CpG probes, multihit probes, and probes located on chromosomes X and Y and (SNP‐related probes) were filtered. The final probes for subsequent analysis were 820 000. Afterward, the β value matrix was normalized using bmiq [11] for adjusting type I and type II probe bias. Next, singular value decomposition analysis (SVD) [12] was then performed to the batch effect caused by BeadChip Slide and Array. combat [12] was applied to correct the batch effect. All CpG sites were annotated using epicanno.ilm10b4.hg19 [13]. Differential methylated CpG positions (probes) were identified using champ. DMP function initiates the limma package to calculate the *P*‐value for differential methylation using a linear model. The DMP function and the adj.*P* values were computed using the Benjamini–Hochberg method [14]. CpGs having |Δβ| ≥ 0.20 and adjusted adj.*P*‐value ≤ 0.05 were considered as DMPs. Differential methylated regions (DMRs) were calculated using probe lasso [15]. All CpGs were for DMR calling, and minSigProbesLasso, lassoRadius, and boundary were the default values. Gene Ontology (GO) [16] and Kyoto Encyclopedia of Genes and Genomes (KEGG) [17] enrichment analysis was conducted using the r package to evaluate the biological processes, cellular components, molecular mechanism, and pathway of DMPs and DMRs. Finally, functional epigenetic modules (FEM) were applied to infer differential methylated gene modules in protein‐to‐protein interaction network (PPI) using the fem [18] package. The copy number variation was also analyzed using the conumee [19] package.

### Pyrosequencing verification

Pyrosequencing is gradually replacing bisulfite sequencing polymerase chain reaction and has become the gold standard for allele quantification in methylation research [20]. Therefore, pyrosequencing was preferred in this study to confirm the methylation level of the 850K array. Ten samples of MG patients were also included for pyrosequencing, besides those used for the 850K array. The sequence of the primers used for PCR after bisulfite conversion was CAMK1D F:TTGGATGATATTAGTAGAGTTGTAGAAA; R:TACCCACCCCTATCTAAAACTACT; S:TGGTTTTTTTTTTATTAGTGAAAAT, and CREB5 F:GGAAAGGTATGGTGAATAAGGTAAATTATG; R:CCAAACTAACAATACCAAAACAACTATAAT; S:TTTTTTTTGTTTTTTATTTTATTTA. The PCR conditions were as follows: 95 °C for 15 min; 94 °C for 30 s; 56 °C for 30 s; 72 °C for 30 s; 72 °C for 10 min (45 cycles). The PCR products were sequenced according to the instructions of the PyroMark Gold Q24 Reagents (Qiagen).

### Cell culture and reagents

Mouse myoblasts C2C12 cells and mouse neuron HT22 cells (Procell Life Science & Technology, Shanghai, China) were cultured in 10% FBS (Gibco, Grand Island, NY, USA) supplemented with 1% penicillin/streptomycin (Gibco). The C2C12 cell culture medium was removed completely at 90% cell confluence, and the DMEM medium containing 2% horse serum was added to induce differentiation of myoblasts. Subsequent experiments were carried out after 5 days of cell differentiation. Cells were incubated at 37 °C in a humidified incubator with 5% CO_2_. The 5‐aza‐2′‐deoxycytidine (DAC) and the procaine hydrochloride were purchased from Sigma (St. Louis, MO, USA).

### Quantitative real‐time PCR

RNA was extracted using the cell total RNA extraction kit (Vazyme Biotechnology, Nanjing, China) and reversely transcribed to cDNA using the HiScript Reverse Transcriptase (Vazyme Biotechnology) according to the manufacturer's instructions. Quantitative detection of mRNA was performed using qPCR CFX96 Optics Module (Bio‐Rad, Hercules, CA, USA). Primers for CAMK1D, CREB5, CALM, PKD, YAP1, and STK3 are listed in Table S1. Gene expression was calculated as $2^{-{\mathrm{\Delta \Delta C}}_{t}}$, with GAPDH mRNA as the internal control.

### Western blot

Cells were first harvested from the culture medium. Subsequent protein extraction was performed on ice for 30 min using RIPA lysis buffer (P0013B; Beyotime, Beijing, China) and protease and phosphatase inhibitor cocktail (P1046; Beyotime). The protein sample was at 13 400 *g* for 30 min at 4 °C. The proteins were concentrated using a BCA kit (MA0082, Meilunbio, Dalian, China), and western blotting was performed as previously described. Primary antibodies against DNMT1 were purchased from Santa Cruz Biotechnology (Santa Cruz, CA, USA). The β‐actin was purchased from Affinity Biosciences (Affinity, Cincinnati, OH, USA). The goat anti‐rabbit IgG and anti‐mouse IgG secondary antibodies were purchased from Affinity Biosciences (Affinity).

## Results

### Patient characteristics

Blood samples from eight MG patients and four healthy controls were collected for Infinium MethylationEPIC BeadChip. The proportion of male‐to‐female patients was 37.5% and 62.5%, respectively. The average age of the study participants was 50 ± 12.9 years old. Three of the eight MG patients previously had MC, while three had MC combined with thymoma (Table 1). After analysis of the sequencing results, 10 MG patients were selected for pyrosequencing verification to confirm the universality of the selected gene methylation changes (Table 1).

**Table 1:** Demographic and clinical characteristics of the samples used in the assay.

Characteristic	MG (*N* = 8) (for methylation sequencing)	MG (*N* = 10) (for pyrosequencing)	Control (*N* = 4)
Sex			
Male (%)	3 (37.5)	5 (50.0)	2 (50)
Female (%)	5 (62.5)	5 (50.0)	2 (50)
Age ± SD	50 ± 12.9	54.9 ± 15.8	42.7 ± 14.5
≤ 50 years (%)	3 (37.5)	4 (40.0)	2 (50)
> 50 years (%)	5 (62.5)	6 (60.0)	2 (50)
Combined with thymoma			
Yes (%)	3 (37.5)	0 (0.0)	0 (0.0)
No (%)	5 (62.5)	10 (100.0)	4 (100.0)
Combined with MG crisis			
Yes (%)	3 (37.5)	0 (0.0)	0 (0.0)
No (%)	5 (62.5)	100 (100.0)	4 (100.0)
Osserman classification			
I (%)	3 (37.5)	4 (40.0)	–
IIa (%)	3 (37.5)	2 (20.0)	–
IIb (%)	1 (12.5)	4 (40.0)	–
III (%)	1 (12.5)	0 (0.0)	–
IV (%)	0 (0)	0 (0.0)	–

### Quality control of genome‐wide methylation detection

**Fig. 1:**
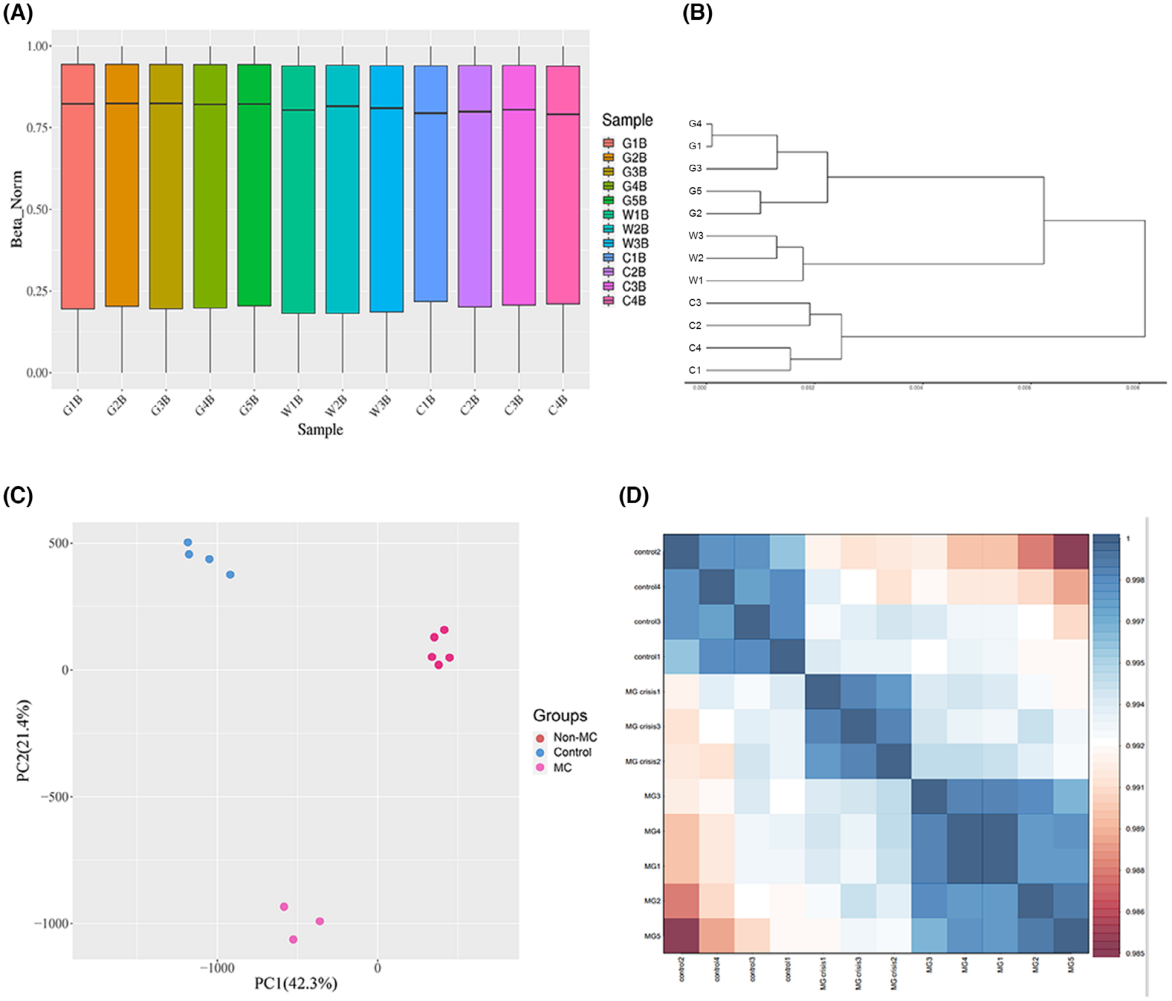
(A) Boxplot used to characterize the overall characteristics of the β value data distribution of each sample, including median value, P25, and P75 quantile value. (B) Sample cluster analysis showing the degree of similarity among all samples. (C) PCA for the normalized beta values of the two groups of samples. PC1, principal component 1; PC2, principal component 2. (D) The sample correlation heat map for two groups of differential methylation sites. The color ranges from red to blue, indicating that the correlation between the two samples is from low to high. The higher the sample correlation, the more similar the overall methylation pattern. Besides, sample G1–G8 represents MG patients in the non‐MC group, W1–W3 represents MG patients in the MC group, and C1–C4 represents the healthy control group.

After quality control of the samples and chips, the median, P25, and P75 quantile values were used to describe the data. The results showed that the values of the experimental samples were evenly distributed, indicating that the data obtained by chip detection were symmetrical (Fig. 1A). CpGs that interfered with subsequent analysis were filtered out using the SVD [21] for quality control on the obtained raw data. The interbatch differences using were corrected using the combat algorithm [12]. Sample cluster analysis mainly showed the degree of similarity among all samples. In the tree graph, the samples were mainly divided into two clusters, with the upper group being MG group. When the cutting height further decreased, the samples were further divided into MC group and the non‐MC group, with the lower group being the control group (Fig. 1B). The principal component analysis (PCA) identified the clusters of samples based on methylation level, which showed the difference between the MG and the control group (Fig. 1C). Similarly, the sample correlation heat map demonstrated the consistency and difference in DNA methylation between those two groups (Fig. 1D). After the homogenization process above, the possible influence of the methylation level variation was eliminated.

### Differential methylation position (DMP) analysis

**Fig. 2:**
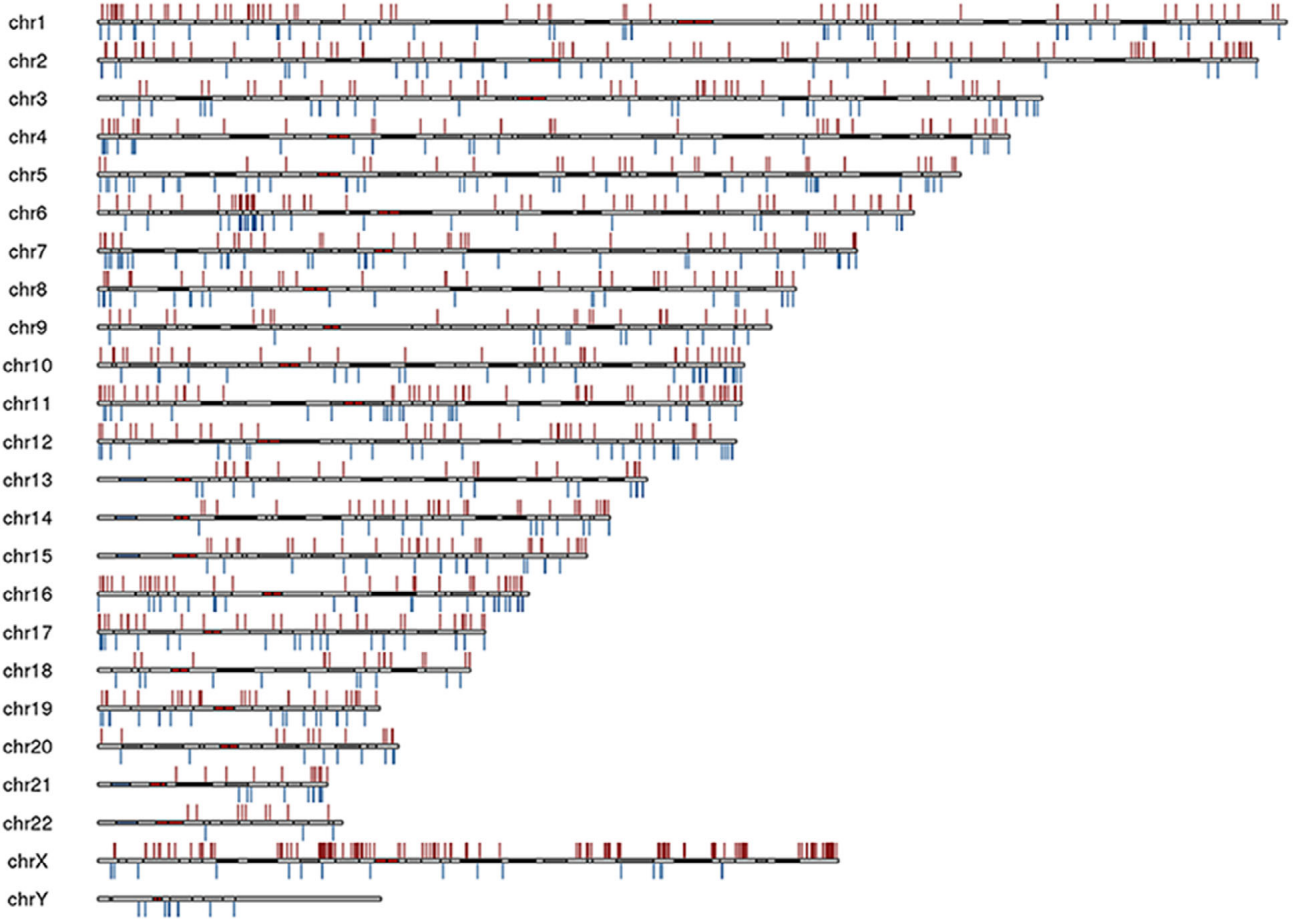
Distribution of differentially methylated positions in the chromosomes in comparison between the MG group and the control group. The red and green bars in the figure represent CpGs with Δβ > 0 (methylation) and Δβ < 0 (hypomethylation), respectively.

The methylation profiles of four comparison groups, control group, MG group, non‐MC group (patients without MC, except control sample), and MC group (patients with MC, except control sample), were compared. A total of 1722 DMPs were screened out, 1091 DMPs were hypermethylated (63.4%), and 631 were hypomethylated (36.6%) in the MG group, compared with controls. Meanwhile, based on the further decrease in cutting height in sample cluster analysis, a difference between MC group and non‐MC group existed. Therefore, the MC and non‐MC groups were further compared, and 2924 DMPs were screened. 1162 (39.7%) DMPs were hypermethylated, and 1762 (60.3%) were hypomethylated (Table 2). Table 2 showed that DMPs located in the body region accounted for a large proportion. Hence, the main focus was the analysis of loci located in the promoter region of functional genes. The comparison between MG and the control group showed that DMPs are widely distributed on chromosomes 1–22 (Fig. 2).

**Table 2:** Distribution of DMPs on functional genomic regions. UTR refers to the untranslated region, Body is between ATG and the stop codon, TSS is the transcription initiation region, TSS1500 refers to 200–1500 bases upstream, TSS200 refers to 0–200 bp upstream of TSS, ExonBnd is located at 20 of the exon boundary within bases.

Functional genomic regions	MG group versus control				MC group versus non‐MC group			
	Hypo DMPs		Hyper DMPs		Hypo DMPs		Hyper DMPs	
	*n*	%	*n*	%	*n*	%	*n*	%
First exon	14	2.2	113	10.4	86	4.9	26	2.2
3′UTR	13	2.1	19	1.7	55	3.1	36	3.1
5′UTR	57	9.0	169	15.5	193	11	97	8.3
Body	267	42.3	391	35.8	644	36.5	503	43.3
TSS1500	59	9.4	141	12.9	231	13.1	111	9.6
TSS200	26	4.1	158	14.5	139	7.9	53	4.6
ExonBnd	4	0.6	2	0.2	7	0.4	4	0.3

**Fig. 3:**
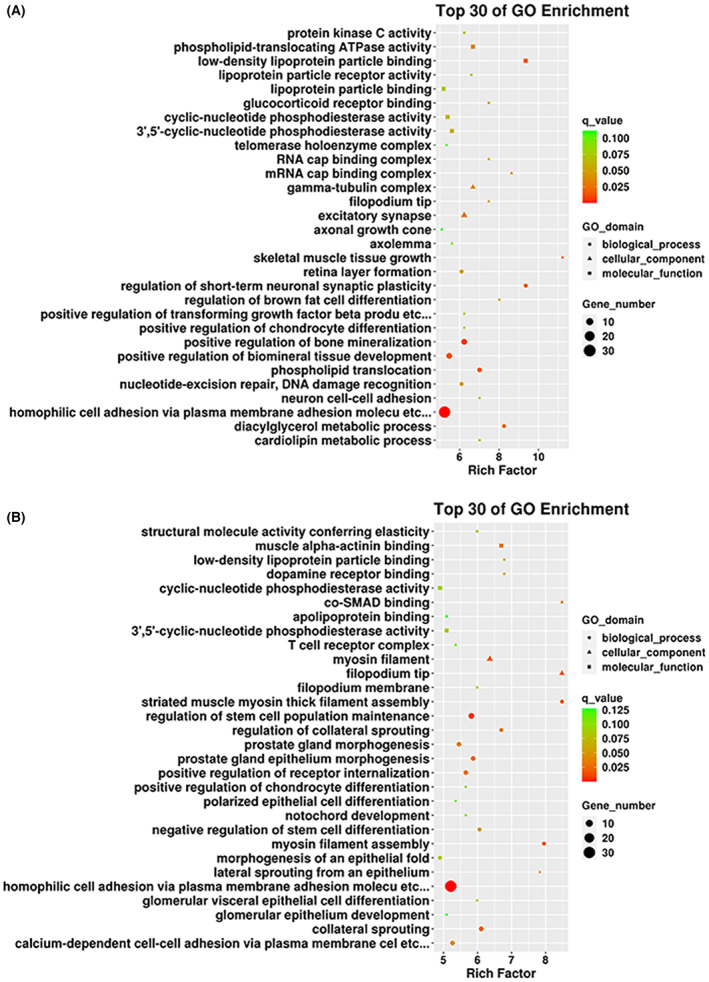
Gene Ontology enrichment analysis of differential methylation sites. (A, B) The top 30 items in the GO enrichment analysis screened after comparing the MG group with the control group, and MC group versus non‐MC group, respectively.

Furthermore, the comparison between the MG and the control group showed that the top 30 GO terms of GO enrichment were enriched in the MG group, including homophilic cell adhesion via plasma membrane adhesion molecules, calcium ion binding, and neuron projection (Fig. 3A). Among the 30 GO terms, eight were related to synapses, and two were related to nerves. This study showed that differentially methylated sites are related to NMJ dysfunction, consistent with MG. The GO enrichment analysis results between MC and non‐MC groups are shown in Fig. 3B. The top 30 GO enrichment items included multicellular organism development and homophilic cell adhesion via plasma membrane adhesion molecules. Of these, four are related to actin and six were related to cell junctions. Actin is one of the structural proteins in muscles. Actin plays an important role in muscle movement and is also consistent with symptoms in MC patients.

**Fig. 4:**
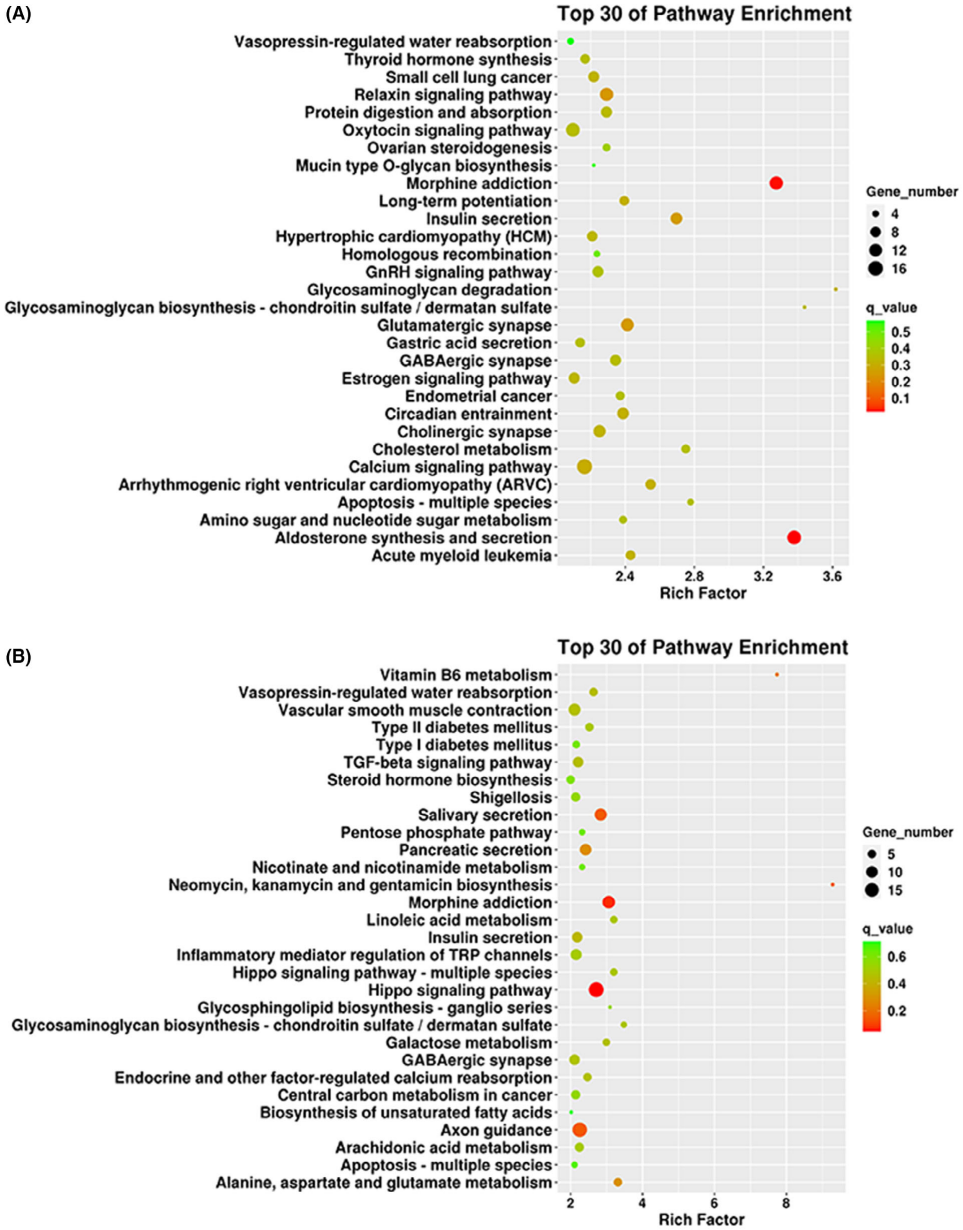
KEGG enrichment analysis of differential methylation sites. (A, B) The top 30 items in the KEGG enrichment analysis screened after comparing the MG group with the control group, and MG MC group versus non‐MC group, respectively.

In addition, 30 enriched pathways were identified after the KEGG analysis of DMP among the MG group compared with the control group. Some of the enriched pathways included aldosterone synthesis and secretion, morphine addiction, and calcium signaling (Fig. 4A). Among the 30 KEGG‐enriched pathways, multiple pathways related to calcium ion channels were found, providing a basis for the subsequent screening of functional genes of abnormal methylation sites in the MG group. The KEGG analysis of the MC versus non‐MC group showed that hippo signaling pathway, morphine addiction, axon guidance, etc. were enriched in the top 30 enrichment pathways (Fig. 4B). Among the top 30 enriched pathways, the hippo signaling pathway showed a significant difference, and this pathway participates in cell death, differentiation, and inhibition of cell proliferation [22]. Clinically, patients with MG often have abnormal thymus, raising the need for subsequent research in this field.

While comparing the MG and control group, 20 key DMPs related to MG were identified, and two key probe sites were selected based on the KEGG results. The corresponding genes were *CAMK1D* and *CREB5* (*P* < 0.05), which were located in the key position of the calcium ion signal pathway. The MC and non‐MC groups were also compared, and 20 important DMPs related to MG were identified, and four key probe sites were selected based on the KEGG results. The corresponding genes were *SAV1, STK3, YAP1*, and *WWTR1* (*P* < 0.05), which were also located in the key position in the hippo signaling pathway.

### Differential methylation region analysis

**Fig. 5:**
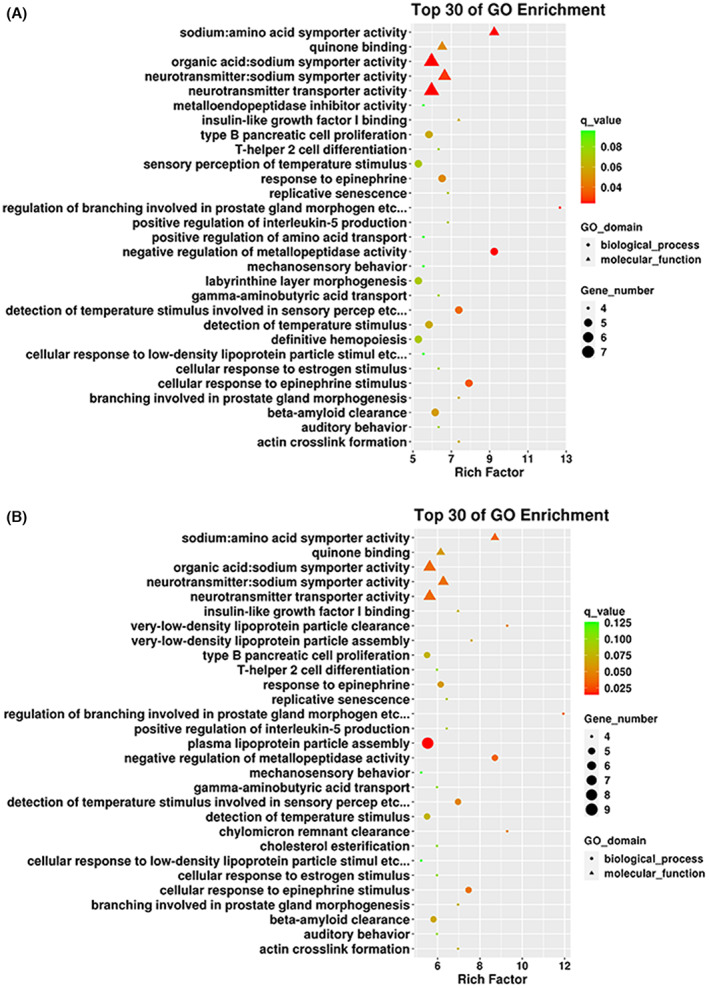
Gene Ontology enrichment analysis of DMR. (A, B) The top 30 items in the GO enrichment analysis screened after comparing the MG group with the control group, and MG MC group versus non‐MC group, respectively.

The MG and control group were compared, and a total of 409 DMRs were filtered in the whole MG patients, such as *HLA‐DQB1*, and other genes of the HLA family were identified in the MG group. Among these 409 DMRs, 116 (28.4%) were located on chromosome 6. According to several studies, chromosome 6 contains the HLA gene, which is associated with autoimmune diseases. The GO enrichment analysis of DMR showed that the GO terms were significantly enriched in neurotransmitter transporter activity, neurotransmitter: sodium symporter and organic acid: sodium symporter activity, which was related to the pathogenesis of MG (Fig. 5A). In addition, we also compared the MC and the non‐MC groups were compared, and 343 DMRs were identified. In addition, plasma lipoprotein particle assembly, neurotransmitter transporter activity, neurotransmitter: sodium symporter, and organic acid: sodium symporter activity also have significant enrichment in the GO enrichment analysis, consistent with the phenomena between the MG and control groups (Fig. 5B).

### Functional epigenetic modules analysis

**Fig. 6:**
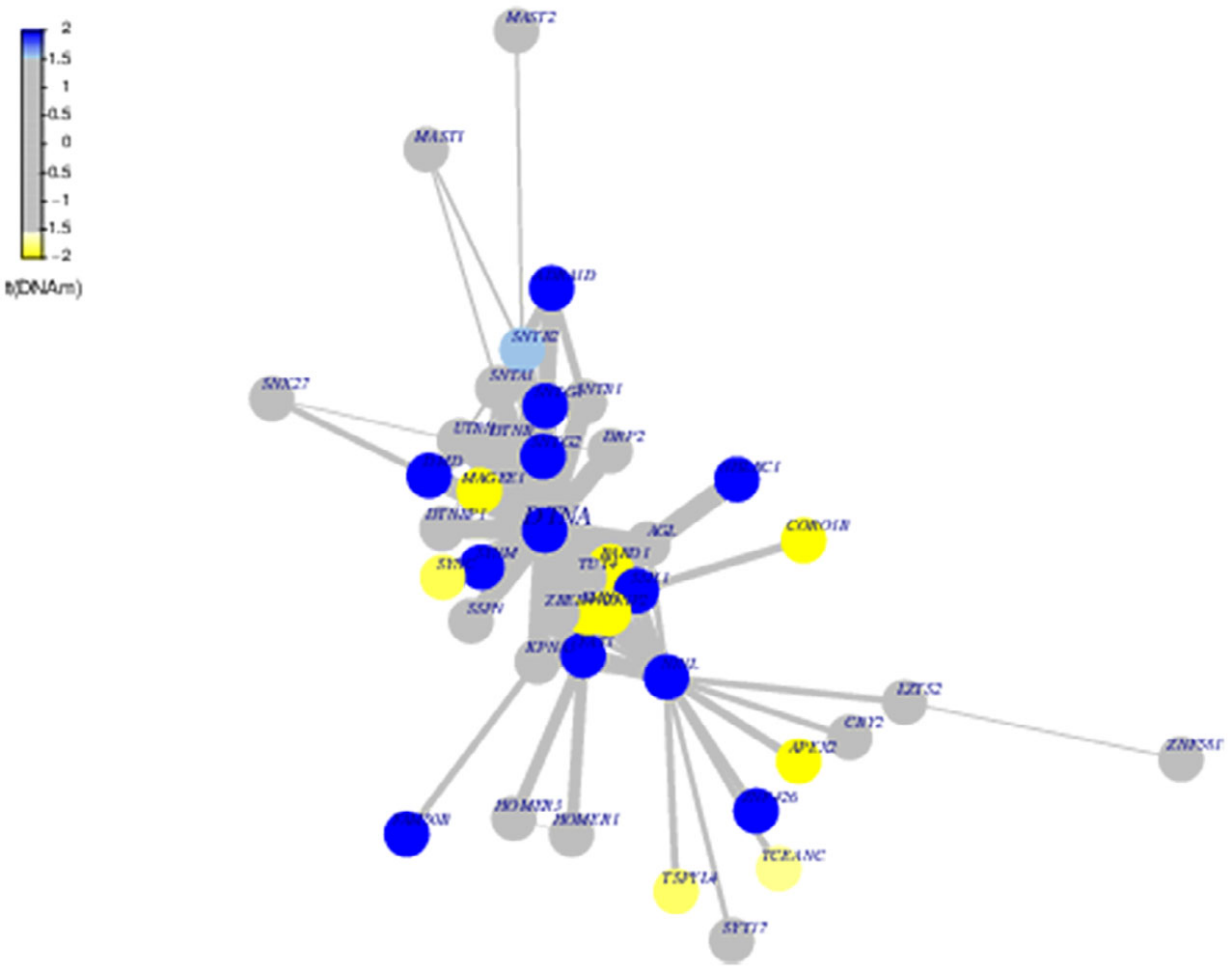
Function epigenetic module display diagram. Each node is a gene, and the color of the node is whether the calculated difference statistic is significant, blue represents positive significance, yellow represents negative significance, and gray represents no difference. The line between nodes is the degree of connectivity between genes, and the thickness of the line represents the level of correlation.

Epigenetics plays an important regulatory role in cell development, cell differentiation, complex disease formation, and tumorigenesis. FEM analysis is a function supervision algorithm that helps identify some genes or proteins with important functions. As shown in Fig. 6, the seed gene of the module was *DTNA*. In addition, *SYNM, SNTG1, SNTG2,* etc. were all positively correlated. The negatively significant genes included *FMN1, MAGEE1*, and *ADNP2. DTNA*, a seed gene, demonstrated its importance in the whole network. According to FEM analysis, the differential modular seed genes and related interacting genes were screened out in the MG and the control group, as well as the comparison between the MC group and non‐MC group.

### Pyrosequencing confirms differences in CAMK1D

**Fig. 7:**
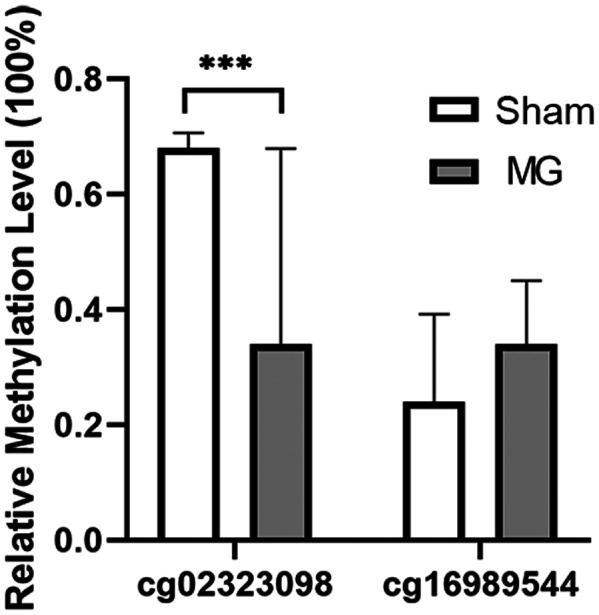
Pyrosequencing confirms differences in CAMK1D. The ordinate shows the relative methylation level, and the abscissa respectively represents the probe cg02323098 of CAMK1D and the probe cg16989544 of CREB. ***P < 0.001. *n* = 10. The data are presented as mean ± standard deviation (SD). Student's *t*‐test was used to compare the two groups.

The current research believes that based on the genome‐wide level of DNA methylation studies, although the sensitivity and breadth have been improved compared with previous techniques, the results obtained still have false‐positive results. During validation, the potential MG differential methylated genes were revalidated using the gold standard for methylation level detection–pyrosequencing technology. The MG and the control groups, and added 10 other samples of the MG group were analyzed based on the existing data. Subsequently, the *CAMK1D* (cg02323098) and *CREB5* (cg16989544) were verified by pyrosequencing. The experimental results showed that the methylation level of *CAMK1D* (cg02323098) in the peripheral blood of MG patients decreased by 52% compared with the control group. The statistical results were significantly different (*P* < 0.001) (Fig. 7).

### Detection of target gene expression in C2C12 cell line and HT22 cell line after demethylating drugs

**Fig. 8:**
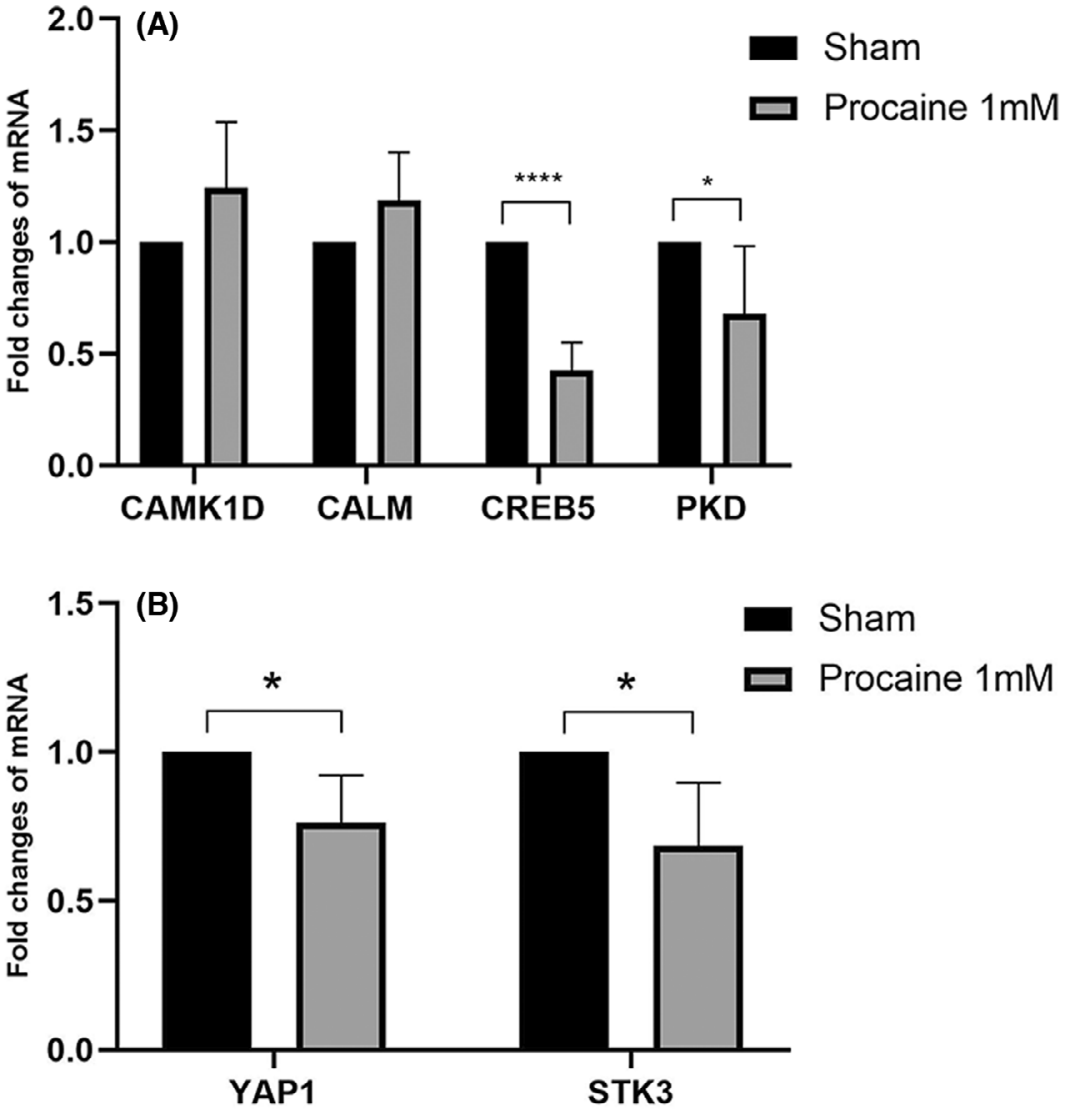
(A) Levels of mRNA expression of CAMK1D, CALM, CREB5, and PKD in C2C12 cell line normalized to those of GAPDH for each sample. (B) The levels of mRNA expression of YAP1 and STK in C2C12 cell line normalized to those of GAPDH for each sample. *P < 0.05, ****P < 0.001 versus the sham group. *n* = 6. The data are presented as mean ± standard deviation (SD). A one‐way ANOVA followed by Fisher's least significant difference was used for statistics.

To further verify the above results, procaine hydrochloride was used to verify demethylation in the C2C12 cell. The mRNA levels of the selected part of the target genes changed significantly, suggesting that the drug treatment was effective. The mRNA levels of *CREB5* and *PKD* were downregulated after procaine hydrochloride [23] treatment compared with the control group (*P* < 0.05) (Fig. 8A). Similarly, compared with the control group, mRNA levels in *YAP1* and *STK3* were also decreased after procaine hydrochloride treatment (*P* < 0.05) (Fig. 8B). No significant difference was observed between *CAMK1D* and *CALM*.

**Fig. 9:**
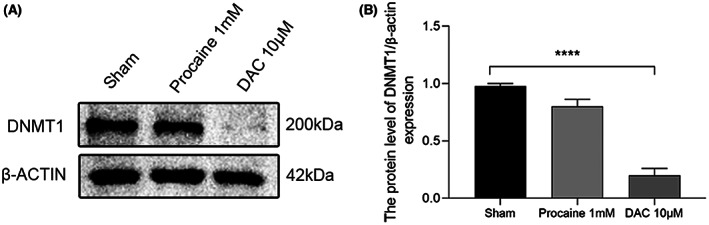
(A) Protein level of DNMT1 expression in HT22 cell line. (B) Analyses of the protein level of DNMT1 expression in HT22 cell line normalized to those of β‐actin for each sample. ****P < 0.0001 versus the sham group. *n* = 4. The data are presented as mean ± standard deviation (SD). A one‐way ANOVA followed by Fisher's least significant difference was used for statistics.

**Fig. 10:**
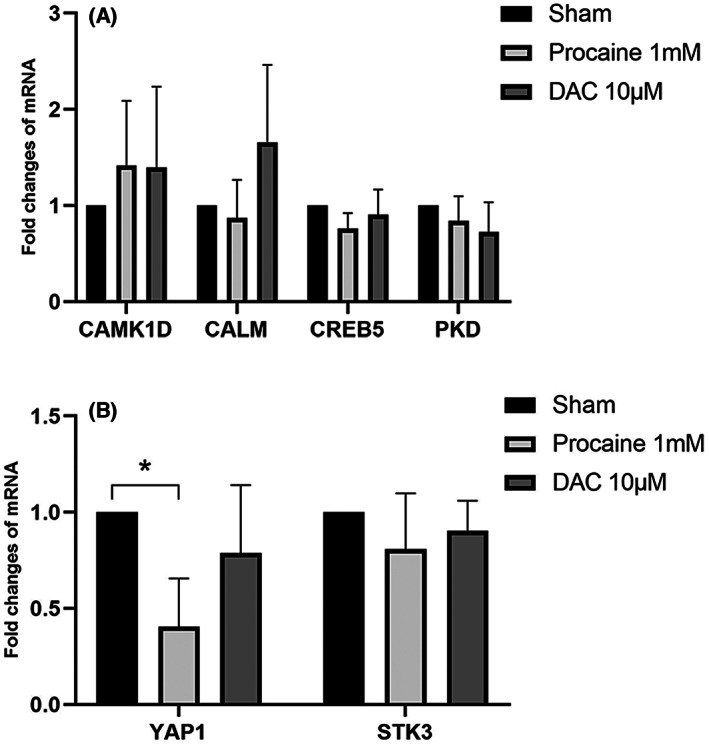
Levels of mRNA expression in HT22 cell line normalized to those of GAPDH for each sample. *P < 0.05 versus the sham group. *n* = 4. The data are presented as mean ± standard deviation (SD). A one‐way ANOVA followed by Fisher's least significant difference was used for statistics.

5‐Aza‐2′‐deoxycytidine [24] and procaine hydrochloride were used for demethylation analysis in HT22 cells. The expression of DNMT1 protein decreased after treatment with the demethylation drugs (Fig. 9A). The experiment showed the expression of DNMT1 was significantly inhibited by DAC (Fig. 9B), indicating that the demethylation effect could be achieved after treatment with demethylation drugs. After 3 days of procaine hydrochloride treatment, only the *YAP1* mRNA transcription was lower after demethylation drugs treatment than in the control group (*P* < 0.05) (Fig. 10B). Conversely, no significant difference was observed in *CAMK1D, CALM, CREB5, PKD*, and *STK3* (Fig. 10A,B).

## Discussion

In this research, the genome‐wide methylation of MG and healthy controls was analyzed using the Infinium MethylationEPIC BeadChip, and the potential methylation sites associated with MG were screened out. Compared with the control group, 1722 DMPs and 409 DMRs were screened out in the MG group. MG is a disorder in the NMJ, *CAMK1D* and *CREB5* were identified, and both were key nodes in the calcium ion signaling pathway using functional annotation. The probes were further validated through pyrosequencing, which showed that the expression of the cg02323098 probe, named *CAMK1D*, was significantly downregulated compared with MG and control groups (*P* < 0.05). Related studies have found that *CAMK1D* is associated with lung adenocarcinoma cells, type 2 diabetes mellitus, and essential hypertension [25, 26, 27]. In addition, some studies have found that *CAMK1D* was a key regulator of tumor innate immune resistance [28]. As an autoimmune disease mediated by autoantibodies, MG can be observed in patients with immunomodulatory defects [29]. However, the ability of *CAMK1D* to regulate this immune response remains to be validated.

The MC and the non‐MC group were compared and screened out 2924 DMPs and 343 DMR. Similarly, GO enrichment and KEGG analysis were performed, and *SAV1, STK3, YAP1,* and *WWTR1*, which were located in the key position of the hippo signaling pathway, were screened out. The hippo signaling pathway is a conservative signal network that regulates tissue homeostasis and organ development. Studies have suggested that the hippo signaling pathway promotes cell death and differentiation, and inhibits cell proliferation [22]. Moreover, the hippo signaling pathway is related to a variety of human diseases, including cancer, autoimmune diseases, and developmental abnormalities [30]. Most AChR^+^ MG patients have thymic changes, 10% have a thymoma, and 80% of the patients with early‐onset MG have thymic follicular hyperplasia [31, 32]. However, whether the changes in the thymus of MG patients are related to the hippo signaling pathway remains to be validated.

In the results, some DMPs/DMRs (such as the presence of low‐level methylation in HLA‐DQB1) have been previously studied and found to be related to the pathogenesis of MG. The past 30 years of research show that the HLA genome contains a large number of genes related to the human immune system. Hence, it is a strong risk factor related to the development of MG [33]. The specific HLA alleles are also thought to be related to the susceptibility of MG [34]. This study is more advantageous because Illumina 850K Beadchips, instead of the Illumina 450K, were used to cover doubled methylation sites. Therefore, more extensive and detailed information was obtained. In the current study, some analyses did not give satisfying results due to the limitation of our small sample size. For example, morphine addiction was observed in the two groups of analysis for the significantly different pathways after KEGG analysis. The above observation lacks a solid explanation for MG patients. Increasing the sample size could validate the results of this study.

Due to the limited number of samples, cell culture experiments were performed further to verify the above findings at the cellular level. The results showed no significant difference in the expression of *CAMK1D* and *CALM* in the C2C12 cell line and the HT22 cell line. However, the expression of the target gene was partially upregulated under treatment with demethylating drugs. Ca^2+^/calmodulin‐dependent protein kinases (CaM‐Ks) regulate various biological events mediated by intracellular Ca^2+^, including muscle contraction, neurotransmitter release, and gene expression [35]. Studies have established *Xenopus* neuromuscular cocultures and found that activation of CaMK is essential for neurotrophic factors to regulate NMJ development [36]. Other studies have found that CaMK mediates the inhibitory effect of BDNF on NMJ maturation, and Ca^2+^ is released from the cell through the IP3 receptor or Ryanodine receptor, regulating the neurotrophic effect of NMJ maturation [37]. Another differential gene, CREB5, was significantly low in C2C12 cell line (*P* < 0.001). The level of *PDK*, an upstream gene was also significantly low (*P* < 0.05). Although no significant difference was observed in HT22 cells, its expression still decreased. As a transcription regulator, *CREB5* is a transcription factor involved in cell survival, cell proliferation, cell adaptation, and differentiation. CREB plays an important role in the immune system by regulating the expression of several inflammatory mediators in white blood cells and macrophages [38, 39, 40].

Due to limited conditions, the pyrosequencing of *YAP1* and *STK3* genes could not be verified in this study. However, following the interest in these genes and their corresponding pathway, the mRNA levels of *YAP1* and *STK3* genes were analyzed at the cellular level. In the C2C12 cell line, the mRNA levels of *YAP1* and *STK3* genes were significantly lower compared with the control group (*P* < 0.05). Similarly, the *YAP1* mRNA level in the HT22 cell line after 3 days of procaine treatment was lower compared with control group (*P* < 0.05). These results are contrary to the expectation of this study. Considering that hippo signaling pathway is a complex signaling pathway, and each key step is affected by multiple factors, the pathway could be regulated by inhibition and promotion factors. However, the nonspecific demethylation drugs used in this study resulted in extensive knockdown of hypomethylation levels, which could not exclude the influence of other genes on the expression of the target gene. However, bioinformatic analysis revealed changes in methylation levels of several key genes of hippo signaling pathway. The hippo signaling pathway is associated with the pathogenesis of several human diseases, including cancer, autoimmune diseases, and developmental abnormalities [30]. The hippo signaling pathway is still a hotspot research area for future studies.

Epigenetic marks are considered one of the important factors related to the development of MG. The screening of methylation molecular markers provides new research ideas and clues for the pathogenesis of MG, and the occurrence and development of the disease.

## Conclusion

850K BeadChips sequencing was performed on the collected MG patients and healthy individuals. The differences in the aldosterone synthesis, secretion, and hippo signaling pathway between MG patients and healthy individuals were determined using GO enrichment and KEGG enrichment analysis. Pyrosequencing analysis confirmed the methylation changes of CAMK1D, which is worthy of further studies. This is the first study to investigate the genome‐scale DNA methylation profiles of MG disease using the Illumina HumanMethylation 850K BeadChip. DNA methylation may be involved in the occurrence and development of MG. It is hoped that through future research, abnormal methylation positions could be used as markers for the clinical screening of MG, screening of related populations, and monitoring MG progression. Thus, methylation analysis has future clinical application potential.

## Conflict of interest

The authors declare no conflict of interest.

### Peer review

The peer review history for this article is available at https://www.webofscience.com/api/gateway/wos/peer‐review/10.1002/2211‐5463.13656.

## Author contributions

BH and PL conceived and designed the project. LT, RZ, and SL acquired the data. JL, LD, CL, and XC analyzed and interpreted the data. JL and SC wrote the paper.

## Supporting information


**Table S1.** Primers used in the polymerase chain reaction.

## Data Availability

Data and analyses will be available from the corresponding author upon reasonable request.
