# Supplementary material for: Epigenome‐wide DNA methylation analysis of myasthenia gravis
Source: FEBS Open Bio. 2023 Jun 10;13(7):1375–89. doi: 10.1002/2211-5463.13656 (PMC10315801; doi:10.1002/2211-5463.13656)
Supplement: Supplementary file 1 — Table S1. Primers used in the polymerase chain reaction. [file FEB4-13-1375-s001.docx]

Table S1: Primers used in the polymerase chain reaction

| Gene | Forward primers | Reverse primers |
| --- | --- | --- |
| CAMK1D | CACCTTCCACGCTCTGTAGTTTCC | GCTTCCAGTGTGCCCTGTTGTC |
| CREB5 | GCAGCGTTGTGATTCAGCAAGC | CAATCTGGCGGTTGGTGGAAGG |
| CALM | TGGTTACATCAGTGCGGCAGAAC | TGACTTGTCCGTCGCCATCAATATC |
| PKD | ATCAGCCGACCCTTTCCCTCAG | CAGAACTTCAGGTGCCAGGTATGC |
| YAP1 | CTAAAGAACCCGAACCGCAGACAG | AGGCAGAACATCCATTATCCAGCAG |
| STK | ACCCACCACCAACATTCAGGAAAC | CTCAGGACTCTTCACCAAGCACTTC |
| GAPDH | AATGTGTCCGTCGTGGATCTGA | AGTGTAGCCCAAGATGCCCTTC |
